# Chitinase 3-like-1 Expression in the Microenvironment Is Associated with Neutrophil Infiltration in Bladder Cancer

**DOI:** 10.3390/ijms242115990

**Published:** 2023-11-05

**Authors:** Ling-Yi Xiao, Yu-Li Su, Shih-Yu Huang, Yi-Hua Chen, Po-Ren Hsueh

**Affiliations:** 1 Department of Laboratory Medicine, China Medical University Hospital, School of Medicine, China Medical University, Taichung 404327, Taiwan; shingloving@gmail.com; 2 Division of Hematology Oncology, Department of Internal Medicine, Kaohsiung Chang Gung Memorial Hospital, Chang Gung University, College of Medicine, Kaohsiung 83301, Taiwan; 3 Genomic & Proteomic Core Laboratory, Department of Medical Research, Kaohsiung Chang Gung Memorial Hospital, Kaohsiung 83301, Taiwan; 4 Division of Infectious Diseases, Department of Internal Medicine, China Medical University Hospital, China Medical University, Taichung 404327, Taiwan; 5 Ph.D. Program for Aging, School of Medicine, China Medical University, Taichung 404327, Taiwan

**Keywords:** bladder cancer, CHI3L1, neutrophil

## Abstract

Bladder cancer is a common cancer with well-established therapeutic strategies. However, recurrence occurs in 50% of patients with non-muscle-invasive bladder cancer, and 20% of patients progress to muscle-invasive bladder cancer. The 5-year survival rate for muscle-invasive bladder cancer patients is disappointingly low, ranging from 36% to 48%. A molecular marker of interest is chitinase 3-like-1 (CHI3L1), which is elevated in various cancers, including bladder cancer. In addition to its role in cancer cells, CHI3L1 also has regulatory abilities in immune cells. Neutrophil infiltration has been shown to positively correlate with overall survival, progression-free survival, and relapse-free survival in bladder cancer patients. However, the relationship between CHI3L1 and neutrophils remain poorly understood. Therefore, this study investigated the relationship between CHI3L1 level and protumor neutrophil infiltration in bladder cancer. We analyzed the GSE128959 dataset and the data of a bladder cancer cohort undergoing chemotherapy. We observed higher expression of CHI3L1 in bladder cancer patients with invasive or chemotherapy-resistance. Our results revealed a positive correlation between CHI3L1 expression and protumor neutrophil infiltration. Elevated CHI3L1 expression was associated with genes which were related to the recruitment and infiltration of neutrophils. Consequently, CHI3L1 may serve as a novel evaluation factor for the degree of neutrophil infiltration in advanced bladder cancer in those scheduled for chemotherapy.

## 1. Introduction

Bladder cancer (BLCA) is a common malignant cancer with a high global recurrence rate. According to the latest data from the GLOBOCAN website, the incidence rate of BLCA is 7.4 per 100,000 people, with mortality and 5-year prevalence rates of 3.7 and 22.1 per 100,000 people, respectively [1]. On the basis of the depth of tissue penetration, BLCA can be categorized into two major types: non-muscle-invasive bladder cancer (NMIBC) and muscle-invasive bladder cancer (MIBC) [2]. Nearly 70% of BLCA patients are diagnosed as having NMIBC, and the remaining 30% present with MIBC. Despite the effectiveness of the transurethral resection of bladder tumors followed by intravesical chemotherapy and Bacille Calmette-Guérin for NMIBC, approximately 50% of patients with NMIBC exhibit recurrence and up to 20% of these patients progress to MIBC [2,3]. Compared with NMIBC, MIBC is associated with increased aggressiveness and higher metastasis and mortality rates [3]. The current standard treatment for MIBC involves neoadjuvant chemotherapy prior to radical cystectomy with pelvic lymph node dissection [2,4]. However, the 5-year survival rate of MIBC remains low, ranging from 36% to 48% [2]. Therefore, delaying the progression or recurrence of NMIBC and improving the prognosis of MIBC are substantial challenges. Precision gene analysis and research are crucial for identifying potential biomarkers that can predict prognosis and survival and thus serve as effective targets for therapy.

Chitinase 3-like-1 (CHI3L1), also known as YKL-40, is a glycoprotein and a member of the 18 glycosyl hydrolase gene family that lacks chitinase activity [5]. CHI3L1 expression has been observed in various cancers, including breast, lung, colon, pancreatic, and even BLCA [6,7,8,9,10]. In addition to being highly expressed in bladder cancer, CHI3L1 expression is also associated with disease-specific survival and metastasis-free survival [10]. Not only is CHI3L1 highly expressed in bladder cancer patient tissues, but higher concentrations of CHI3L1 are also detected in patient serum. Patients with higher serum CHI3L1 concentrations also have relatively poorer disease-free survival after surgery [11]. Additionally, CHI3L1 can promote bladder cancer cell migration and invasion by regulating EMT gene expression [12]. In addition to the cancer cells themselves, there are also many other cells that promote tumor progression, such as immune cells, fibroblasts, stromal cells, etc. [13]. CHI3L1 regulates the reprogramming of macrophages, development of CD4^+^ T cells, and cytotoxic activity of CD8^+^ T cells during cancer progression [14,15,16]. Moreover, the secretion of CHI3L1 by macrophages, CD4^+^ T cells, and CD8^+^ T cells fosters an immunosuppressive tumor microenvironment [17]. However, in addition to the aforementioned T cells and macrophages, neutrophils also participate in tumor progression within the tumor tissue. The degree of neutrophil infiltration in the tumor area of MIBC patients is higher than that of NMIBC patients. Meanwhile, bladder cancer patients with high degrees of neutrophil infiltration also have poorer relapse-free survival and overall survival (OS) [18]. Additionally, the number of tumor-infiltrating neutrophils is significantly increased in high-grade or muscle-invasive bladder cancer patients [19]. Apart from being associated with bladder cancer patient staging and survival, the degree of neutrophil infiltration in the tumor microenvironment is also positively correlated with lymph node metastasis [20]. Although it is known that CHI3L1 can create a tumor-promoting environment by regulating T cells and macrophages, its association with neutrophils has only been explored in liver inflammation and cystic fibrosis [21,22]. Even though CHI3L1 and neutrophil infiltration have each been implicated in bladder cancer patient survival, the correlation between these two factors has not yet been explored or understood in bladder cancer research.

In this study, we investigated the relationship between CHI3L1 expression and neutrophil infiltration in bladder cancer. We evaluated the expression of CHI3L1 along with clinical parameters and neutrophil infiltration by using the publicly available dataset (GSE128959) and a bladder cancer cohort undergoing chemotherapy. In summary, CHI3L1 may serve as an assessment factor for evaluating neutrophil infiltration status in bladder cancer.

## 2. Results

### 2.1. CHI3L1 Expression Is Associated with a Poor Prognosis and Advanced Stage in BLCA

**Figure 1:**
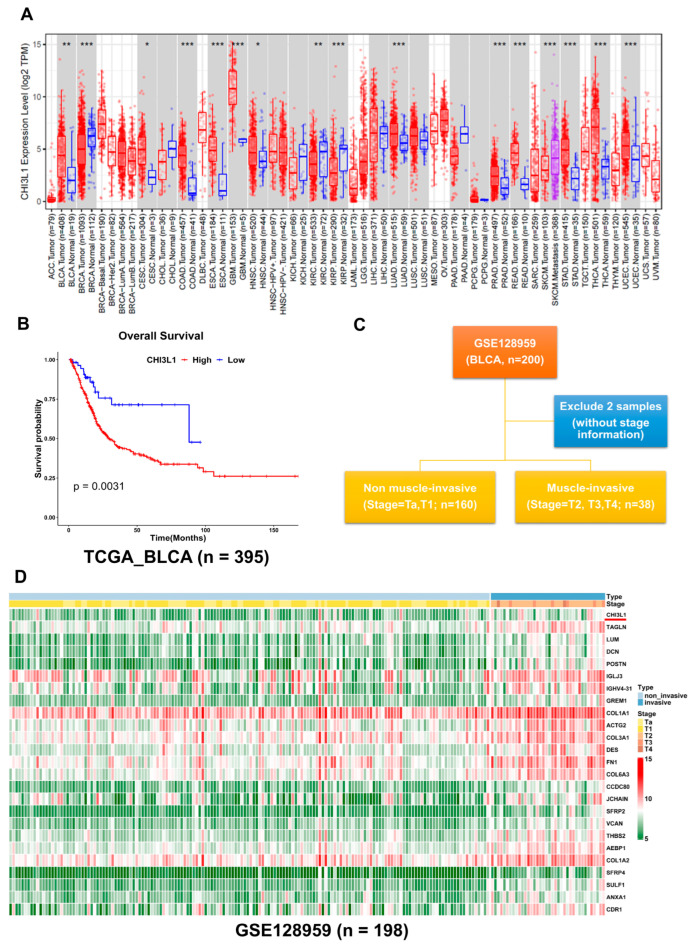
CHI3L1 overexpression is associated with poor prognosis and invasive BLCA patients. (**A**) The CHI3L1 level in different cancer types from the TCGA database in TIMER (red: tumor; blue: normal) (* *p* < 0.05, ** *p* < 0.005, *** *p* < 0.001). (**B**) Kaplan–Meier method was used to examine OS in patients with BLCA in TCGA (*p* = 0.0031). (**C**) The analyzed diagram of the GSE128959 dataset. (**D**) Heatmap of significant differentially expressed genes in the non-invasive group and invasive group of the GSE128959 dataset.

To investigate the potential role of CHI3L1 in BLCA, we initially analyzed its expression in various cancer types by using data from the Tumor Immune Estimation Resource (TIMER). As illustrated in Figure 1A, we observed high CHI3L1 expression in several cancers, including BLCA, esophageal carcinoma, lung adenocarcinoma, and thyroid carcinoma. According to TCGA data, among patients with BLCA, those with high CHI3L1 expression had a shorter OS than did those with low CHI3L1 expression. The median OS was 29.1 months in the high CHI3L1 expression group but 88.8 months in the low CHI3L1 expression group (Figure 1B). To validate the role of CHI3L1 in BLCA, we analyzed the GSE128959 dataset from the Gene Expression Omnibus (GEO), which consisted of 200 formalin-fixed paraffin-embedded samples from patients diagnosed as having BLCA. However, two samples had missing stage information. Thus, only 198 samples were included in the subsequent analysis. For the analysis of differentially expressed genes, these samples were divided into two groups based on the diagnosed clinical stage: non-invasive (stage Ta, T1; *n* = 160) and invasive (stages T2, T3 and T4; *n* = 38) (Figure 1C). We selected the top 30 genes with fold changes greater than 1.15 and *p*-values less than 0.05 on the heatmap. The heatmap revealed the elevated expression of CHI3L1 in the invasive group than in the non-invasive group (Figure 1D). We also used scatter plots to visualize the expression level of CHI3L1 in non-invasive and invasive samples from the GSE128959, and we confirmed a significant increase in CHI3L1 expression in the invasive samples (Supplementary Figure S1, *p* < 0.0001). These findings demonstrate that CHI3L1 expression is not only elevated but also associated with poorer OS and advanced disease stage in patients with BLCA.

### 2.2. CHI3L1 Expression Is Correlated with Neutrophil Infiltration and Protumor Neutrophil Markers in BLCA

**Figure 2:**
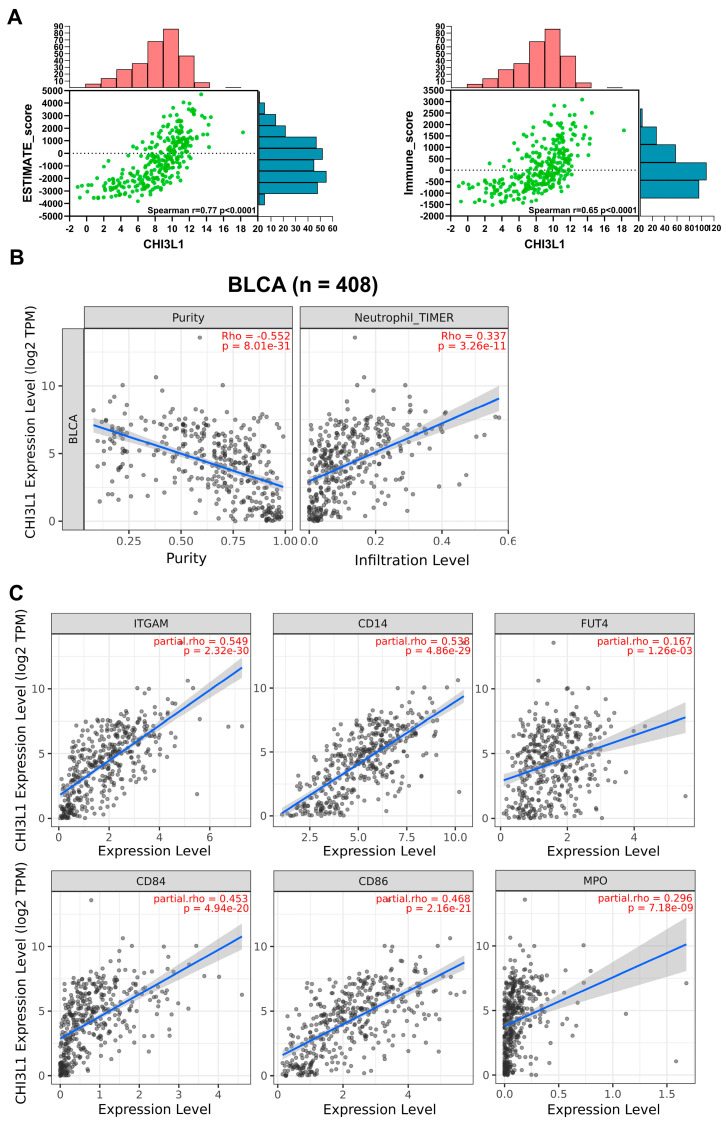
CHI3L1 expression is associated with neutrophil infiltration and protumor neutrophil markers in BLCA. (**A**) The relationship between CHI3L1 and ESTIMATE and immune scores. (**B**) The correlation between neutrophil infiltration and CHI3L1 expression. (**C**) The correlation between CHI3L1 expression and protumor neutrophil markers (CD11b, CD14, CD15, CD84, CD86, and MPO). All scatter plots were statistically significant, *p* < 0.05.

Previous studies have demonstrated that the neutrophil-to-lymphocyte ratio and baseline neutrophil count are associated with the prognosis of patients with MIBC undergoing chemoradiation treatment [23,24]. Furthermore, activated neutrophils have been observed to suppress liver inflammation by secreting CHI3L1 [22]. Moreover, CHI3L1 is involved in the creation of an immuno-suppressive tumor microenvironment. Therefore, in this study, we examined the association between CHI3L1 expression and immune cell infiltration, particularly neutrophil infiltration, in patients with bladder cancer by using the ESTIMATE algorithm and TIMER algorithm. According to the result of the ESTIMATE algorithm, CHI3L1 expression was positively correlated with the ESTIMATE score (r = 0.77, *p* < 0.0001) and the Immune score (r = 0.65, *p* < 0.0001; Figure 2A). In addition, CHI3L1 expression displayed a negative correlation with tumor purity (Rho = −0.552, *p* = 8.01 × 10^−31^) but a significant positive correlation with neutrophil infiltration (Rho = 0.337, *p* = 3.26 × 10^−11^; Figure 2B). In addition to using the TIMER algorithms, we also employed the CIBERSORT and CIBERSORT-ABS algorithms to further validate the relationship between CHI3L1 and neutrophil infiltration. Supplementary Figure S2A illustrates that CHI3L1 expression was positively correlated with neutrophil infiltration. In addition to neutrophils, we further investigated the correlation between CHI3L1 and other immune cells including CD4^+^ T cells, CD8^+^ T cells, macrophages, and dendritic cells (Supplementary Figure S2B). We observed that the positive correlation also existed in CHI3L1 and other immune cells. Furthermore, we analyzed the relationship between CHI3L1 expression and neutrophil-associated markers contributing to cancer progression [25,26,27]. The results revealed significant positive correlations between CHI3L1 expression and the immunosuppression-related neutrophil markers CD11b (ITGAM), CD84, and CD86. In addition, CHI3L1 expression was positively correlated with the neutrophil markers CD14, CD15 (FUT4), and MPO, which promote tumor cell invasion and proliferation (Figure 2C). In summary, the elevated expression of CHI3L1 in BLCA is related with protumor neutrophil infiltration.

### 2.3. CHI3L1-Related Genes Are Positively Correlated with Neutrophil Infiltration and Protumor Neutrophil Markers

**Figure 3:**
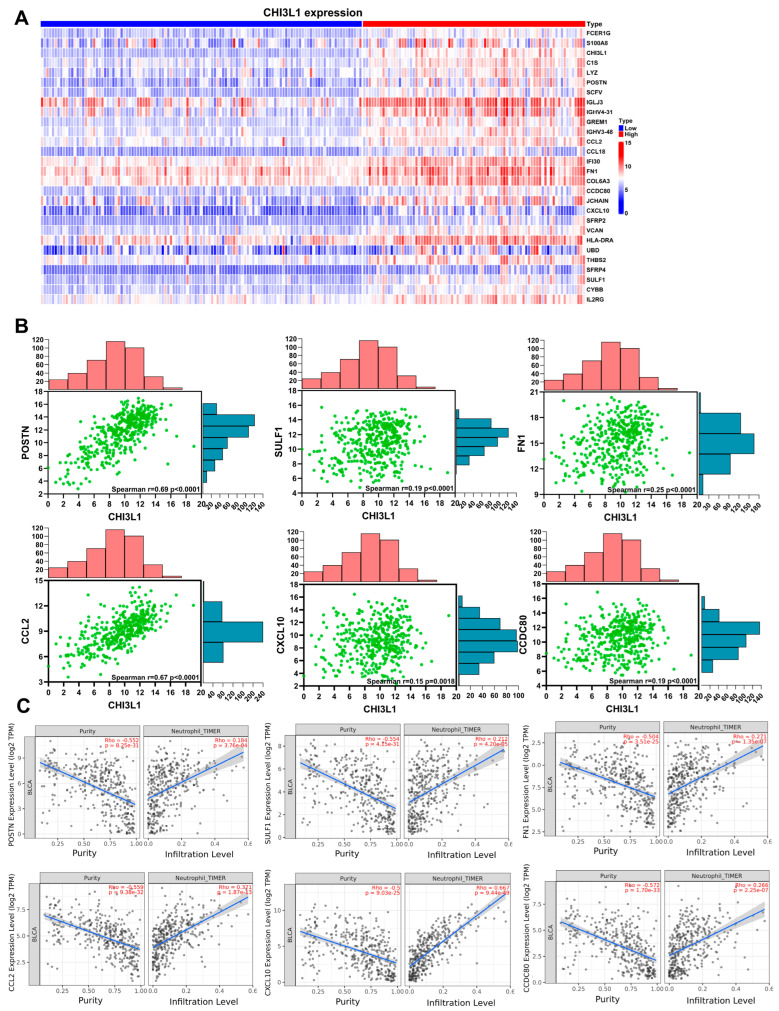
CHI3L1-related genes are correlated with neutrophil infiltration in BLCA. (**A**) Heatmap of significant differentially expressed genes in the high-level and low-level CHI3L1 groups from the GSE128959 dataset. (**B**) The correlation between CHI3L1 and differentially expressed genes (*POSTN*, *SULF1*, *FN1*, *CCL2*, *CXCL10*, and *CCDC80*). (**C**) The correlation between CHI3L1-related genes (*POSTN*, *SULF1*, *FN1*, *CCL2*, *CXCL10*, and *CCDC80*) expression and neutrophil infiltration.

To identify the specific molecules involved in CHI3L1-mediated effects on neutrophils in BLCA, we conducted a subgroup analysis by using the GSE128959 dataset which was divided into a high CHI3L1 expression group and a low CHI3L1 expression group. The heatmap depicted in Figure 3A displayed the genes with log-fold changes greater than 1.1 and *p*-values less than 0.05. We analyzed the correlation between CHI3L1 expression and differentially expressed genes that are identified through literature searches as known to influence neutrophil recruitment or infiltration in the TCGA_BLCA dataset. These genes include *POSTN*, *SULF1*, *FN1*, *CCL2*, *CXCL10*, and *CCDC80*. The results revealed that CHI3L1 expression was positively correlated with the expression of two chemokine genes, *CCL2* (r = 0.67, *p* < 0.0001) and *CXCL10* (r = 0.15, *p* = 0.0018). Moreover, CHI3L1 exhibited positive correlations with genes associated with neutrophil infiltration, including *POSTN* (r = 0.69, *p* < 0.0001), *SULF1* (r = 0.19, *p* < 0.0001), *FN1* (r = 0.25, *p* < 0.0001), and *CCDC80* (r = 0.19, *p* < 0.0001; Figure 3B). We investigated whether these CHI3L1-related genes are associated with neutrophil infiltration by analyzing the data from TIMER. As illustrated in Figure 3C, the chemokine genes (*CCL2* and *CXCL10*) and neutrophil infiltration-related genes (*POSTN*, *SULF1*, *FN1*, and *CCDC80*) were correlated with neutrophil infiltration. We determined that all six of these correlated genes (*POSTN*, *SULF1*, *FN1*, *CCL2*, *CXCL10,* and *CCDC80*) were positively correlated with protumor neutrophil markers, including CD11b (ITGAM), CD14, CD15 (FUT4), CD84, CD86, and MPO (all *p* < 0.05; Supplementary Figure S3). Therefore, the genes that were positively correlated with CHI3L1 expression were associated with neutrophil infiltration and protumor neutrophil markers.

### 2.4. CHI3L1 Is Overexpressed in Patients with Chemotherapy Resistant Bladder Cancer

**Figure 4:**
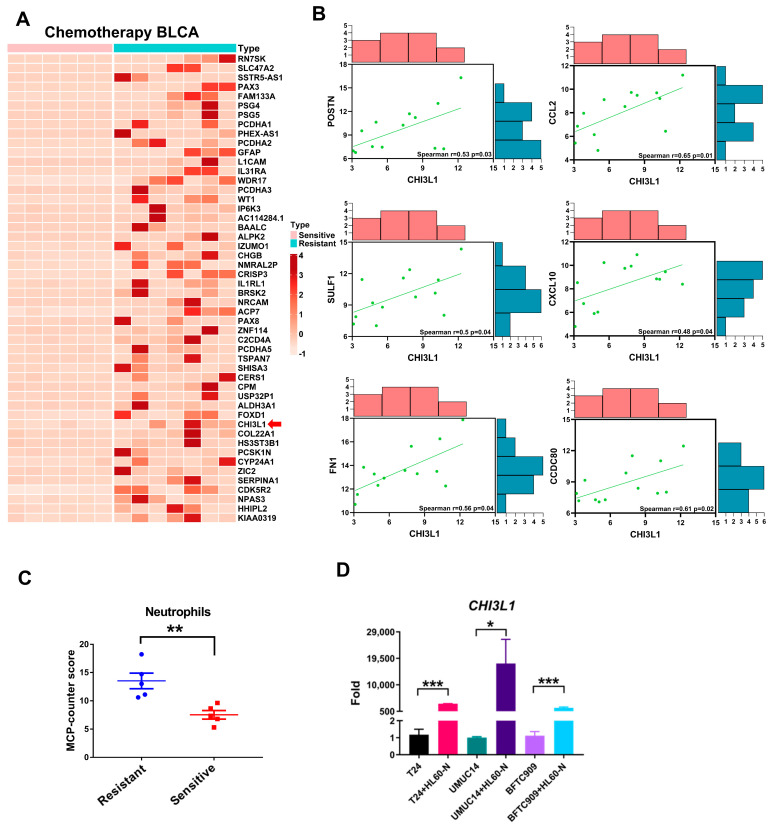
Patients with chemotherapy resistant bladder cancer exhibit a high level of CHI3L1 expression and neutrophil infiltration. (**A**) Heatmap of up-regulated differentially expressed genes in the chemotherapy-sensitive and chemotherapy-resistant groups. (**B**) The correlation between CHI3L1-related genes (*POSTN*, *SULF1*, *FN1*, *CCL2*, *CXCL10*, and *CCDC80*) and CHI3L1 expression in the chemotherapy BLCA cohort. (**C**) The neutrophil count was compared between chemotherapy-resistant and chemotherapy-sensitive patients (** *p* < 0.01). (**D**) The differences of CHI3L1 mRNA expression while bladder cancer cells (T24, UMUC14, BFTC909) were co-cultured with or without HL60-N cells (* *p* < 0.05, *** *p* < 0.002).

In addition to the connection between CHI3L1 and neutrophil infiltration in patients with advanced BLCA, we further wanted to know whether CHI3L1 expression was linked to neutrophil infiltration and chemotherapy sensitivity in BLCA patients. We analyzed a cohort of 13 BLCA patients undergoing chemotherapy, with 6 and 7 patients classified as being sensitive and resistant to chemotherapy, respectively. By comparing differentially expressed genes between these groups, we identified significant genes exhibiting significantly higher expression in the chemotherapy resistant patients, and CHI3L1 was among these genes (Figure 4A). We further visually represented the CHI3L1 expression values for each patient, confirming that patients resistant to chemotherapy indeed exhibited higher CHI3L1 expression (Supplementary Figure S4, *p* < 0.05). Similarly, we examined the correlation between CHI3L1 and CHI3L1-related genes in this chemotherapy-treated cohort. In this cohort, CHI3L1 was significantly correlated with POSTN, SULF1, FN1, CCL2, CXCL10, and CCDC80 (Figure 4B). Furthermore, we observed that chemotherapy-resistant patients had higher microenvironment cell populations counter-derived neutrophil values for neutrophils than did chemotherapy-sensitive patients, with a *p*-value of 0.008 (Figure 4C). Moreover, HL60 cells were stimulated to differentiate into neutrophil-like cells (HL60-N) (Supplementary Figure S5). Subsequently, the expression level of CHI3L1 mRNA significantly increased when bladder cancer cells (T24, UMUC14, BFTC909) were co-cultured with HL60-N cells (Figure 4D). These findings indicated that CHI3L1 expression was positivity related with neutrophil infiltration in chemotherapy resistant patients. In addition, neutrophils had the ability to regulate CHI3L1 expression in bladder cancer.

## 3. Discussion

BLCA is a major global health concern, and the identification of prognostic biomarkers and therapeutic targets is crucial to improve patient outcomes. In this study, we investigated the relationship between CHI3L1 expression and neutrophils in BLCA. We performed a comprehensive analysis by using public datasets and data from a cohort of BLCA patients undergoing chemotherapy. Our findings revealed the association of CHI3L1 expression with invasive or chemotherapy-resistant bladder cancer. In addition, CHI3L1 expression was positively correlated with neutrophil infiltration and protumor neutrophil markers. Moreover, a positive correlation was noted between CHI3L1 expression and the genes of neutrophil recruitment and infiltration, namely *POSTN*, *SULF1*, *FN1*, *CCL2*, *CXCL10*, and *CCDC80*. In conclusion, our results revealed the substantial role of CHI3L1 in BLCA and its association with protumor neutrophil infiltration.

Despite our observation of a higher expression of CHI3L1 in patients with invasive or chemotherapy-resistant bladder cancer and the association of CHI3L1 with increased neutrophil infiltration, the cellular sources of CHI3L1 secretion in the cancer microenvironment remain elusive. Additionally, the mechanism through which CHI3L1 regulates neutrophil infiltration, which affects tumor progression and the drug response in BLCA patients, remains unclear. In colorectal cancer, cancer-associated fibroblasts secrete CHI3L1 to regulate tumor angiogenesis [28]. In breast and gastric cancer, CHI3L1 can be secreted by cancer cells or macrophages, affecting tumor metastasis [29,30]. CHI3L1 secretion by cancer stem-like cells leads to the inhibition of drug-induced apoptosis in ovarian cancer [31]. Therefore, various cell types within the tumor microenvironment can enhance CHI3L1 expression in patients with invasive or chemotherapy-resistant bladder cancer. Further studies should explore the cellular origin of CHI3L1 secretion in bladder cancer and elucidate how it leads to increased neutrophil infiltration.

In our experimental results obtained using the GSE128959 dataset and the data of the chemotherapy-treated BLCA cohort, we observed a positive correlation between the expression levels of CHI3L1 and POSTN (Figure 3 and Figure 4). POSTN plays a unique role as an inducer of chemokines that recruit neutrophils [32]. Additionally, we noted positive correlations between CHI3L1 and the chemokine genes *CCL2* and *CXCL10*, both of which are involved in the migration of neutrophils to inflammation sites. In acute respiratory distress syndrome, CCL2 and CXCL10 affect the recruitment of neutrophils [33,34]. Moreover, neutrophil migration and recruitment to tumor sites are mediated by the CCL2–CCR2 axis [35]. Similarly, CXCL10 binds to its receptor, CXCR3, for the recruitment of neutrophils [36]. These findings indicate that high CHI3L1 expression may affect the secretion of chemokines (CCL2 and CXCL10) through POSTN, ultimately leading to increased neutrophil infiltration. However, the detailed mechanisms underlying this process should be elucidated to better understand the regulatory role of CHI3L1 in neutrophil infiltration in BLCA. In chemotherapy-induced interstitial lung disease, increased POSTN expression is accompanied by neutrophil recruitment [32]. Therefore, future studies should determine whether CHI3L1, through its regulation of POSTN expression and function, affects the response of patients with bladder cancer to chemotherapeutic drugs.

Our results revealed that three other genes, *SULF1*, *FN1*, and *CCDC80*, are positively correlated with CHI3L1 (Figure 3 and Figure 4). Previous studies have demonstrated a close relationship between these genes and immune cell infiltration in various cancers, including gastric cancer, thyroid carcinoma, breast cancer, and colorectal cancer [37,38,39]. Nevertheless, it remains unclear whether these genes independently regulate neutrophil infiltration or act in collaboration with CHI3L1. Furthermore, although we observed a positive correlation between CHI3L1 expression and neutrophil infiltration by using the GSE128959 dataset and the data of the chemotherapy BLCA cohort, the regulatory mechanisms underlying the effect of neutrophil infiltration on BLCA progression or drug response remain unclear. Future studies should elucidate these mechanisms to gain a better understanding of the functions of CHI3L1. This knowledge can provide clinical insights for assessing the factors contributing to progression of BLCA patients toward an invasive phenotype and determining patients’ suitability for chemotherapy.

In addition to neutrophils, we also observed positive correlations between CHI3L1 and infiltration of other immune cells, including CD4^+^ T cells, CD8^+^ T cells, macrophages, and dendritic cells. Remarkably, the correlation with dendritic cells (Rho = 0.371) exceeded that with neutrophils (Rho = 0.337) (Figure 2B, Supplementary Figure S2B). Dendritic cells have the capacity to attract and retain neutrophils through the secretion of IL-8 [40,41]. Therefore, it is plausible that CHI3L1 might enhance neutrophil infiltration by promoting the recruitment of dendritic cells. Conversely, it is important to note that neutrophils also secrete chemokines that attract dendritic cells [42,43]. Therefore, there may be a positive feedback loop between dendritic cells and neutrophils mediated by CHI3L1. This intricate interplay may contribute to escalated infiltration, fostering an immunosuppressive environment and, ultimately, disease progression and poorer prognosis in bladder cancer patients. It is imperative to conduct further in-depth investigations to elucidate and validate these speculations in future studies.

Tumor-associated neutrophils (TANs) exhibit protumor effects including promoting immunosuppression, tumor cell proliferation, angiogenesis, and metastasis [44,45,46]. Moreover, tumor-infiltrating neutrophils (TINs) play a crucial role in tumor development, progression, and resistance to therapy in numerous cancers [47,48]. Notably, in MIBC, the level of TANs serves as a predictive factor for shorter relapse-free survival and poorer OS [18]. The standard treatment for MIBC involves cisplatin-based combination chemotherapy [49,50], and MIBC patients with low TIN levels benefit from adjuvant chemotherapy [51]. In our study, we observed that the expression pattern of CHI3L1 significantly influences neutrophil infiltration in MIBC patients. Furthermore, bladder cancer patients with chemotherapy resistance tend to have higher neutrophil counts. Therefore, for MIBC patients, the expression level of CHI3L1 may serve as an evaluation factor for TANs infiltration, as well as a marker for therapeutic efficiency and prognosis.

## 4. Materials and Methods

### 4.1. Tumor Immune Estimation Resource (TIMER)

TIMER (http://timer.cistrome.org/, accessed on 1 January 2023) is a web-based tool used for the comprehensive assessment of immune cell infiltration in various cancer types. We used TIMER to analyze CHI3L1 expression in multiple cancer types and to examine the correlation between CHI3L1 and neutrophil infiltration and protumor neutrophil markers.

### 4.2. The Cancer Genome Atlas (TCGA) and cBioPortal

Gene expression data and clinical information from BLCA samples were obtained from cBioPortal (https://www.cbioportal.org/, accessed on 3 February 2023) and TCGA (https://tcga-data.nci.nih.gov/taga/, accessed on 3 February 2023) by using R software (version R4.2.1). We analyzed CHI3L1 expression data in relation to survival outcomes and relevant genes in BLCA.

### 4.3. Public Transcriptome Analysis

The dataset GSE128959 from the Gene Expression Omnibus (GEO) (https://www.ncbi.nlm.nih.gov/geo/, accessed on 1 January 2023) database was downloaded and analyzed using R software (version R4.2.1). For data analysis, significantly and differentially expressed genes were selected based on the criteria of logFoldChange > 1.15 and *p* < 0.05.

### 4.4. Estimation of STromal and Immune Cells in MAlignant Tumours Using Expression Data (ESTIMATE) Score and Immune Score

The ESTIMATE algorithm (https://bioinformatics.mdanderson.org/estimate/, accessed on 17 May 2023) was used to calculate the ESTIMATE and immune scores. The ESTIMATE, immune, and stromal scores were calculated for each sample. The ESTIMATE score is defined as the combination of immune and stromal scores to infer tumor purity [52].

### 4.5. Patient Samples and Clinical Information

The specimens of BLCA patients were collected after obtaining approval from the Institutional Review Board (IRB) of Chang Gung Memorial Hospital (IRB number 201901981B0). RNA was extracted from the samples and subjected to RNA sequencing. Using fragments per kilobase per million (FPKM) data, we examined differentially expressed genes with R software (version R4.2.1), considering parameters such as log2FoldChange > 2 and *p* < 0.05.

### 4.6. Cell Culture and Differentiation

Human bladder cancer cell lines (T24, UMUC14 and BFTC909) and promyelocytic leukemia cells (HL60) were supplemented with basal medium, 10% FBS and 1% Penicillin/Streptomycin (Gibco, Thermo Fisher Scientific, Waltham, MA, USA) in a humidified atmosphere containing 5% CO_2_ at 37 °C. DMEM medium was used for UMUC14 and BFTC909 cells. Myco’5A medium was used for T24 cells, and RPMI1640 medium was used for HL60 cells (Gibco, Thermo Fisher Scientific, Waltham, MA, USA). In addition, 1.3% DMSO (Sigma, Saint Louis, MO, USA) was used to induce the differentiation of HL60 cells from neutrophil-like cells (HL60-N). The expression of neutrophil surface markers (CD11b and CD16) was analyzed using BD LSRII (BD Biosciences, San Jose, CA, USA), and the results were analyzed using the FlowJo V10 software. The fluorescence for CD11b was labeled with APC, and for CD16, it was labeled with FITC (Biolegend, San Diego, CA, USA) [53].

### 4.7. Real-Time Reverse Transcriptase-Polymerase Chain Reaction

T24, UMUC14, and BFTC909 cells were cultured with or without HL60-N cells for 48 h. For the co-culture experiment, the ratio between bladder cancer cells and HL60-N cells was 1:3. Total RNA from bladder cancer cells was extracted and extracted RNA was reverse-transcribed to cDNA. The RNA expression level of CHI3L1 and GAPDH was performed by using a QuantiNova SYBR Green kit (QIAGEN, Hilden, Germany) and ABI7500 Fast Real-Time PCR system (Applied Biosystems, Thermo Fisher Scientific, Waltham, MA, USA). The running protocol was 95 °C for 2 min, followed by 40 cycles of 95 °C for 2 s and 60 °C for 10 s. Each assay was performed in triplicate.

### 4.8. Statistical Analysis

R software was used to perform statistical analysis and generate figures. Survival analysis was performed using the “Survminer” and “Survival” R packages. Retrieval from TCGA and cBioportal was performed using the “TCGAbiolinks” and “gdsr” R packages. Differentially expressed genes were analyzed using the “limma” and “DESeq2” R packages. Data visualization was performed using R packages that included “ggplot2”, “ggstatsplot”, ”ggpubr” and “ComplexHeatmap”. Spearman correlation analysis was performed to examine the relationship between different genes. MCP counter scores were examined using GraphPad Prism 9, and Student’s two-tailed *t* test was used. In all statistical analyses, a *p* value of <0.05 was considered statistically significant.

## Data Availability

The mRNA expression data used in this study are deposited in the GEO repository (GSE128959). (https://www.ncbi.nlm.nih.gov/geo/query/acc.cgi?acc=GSE128959) (accessed on 1 January 2023).

## Supplementary Materials

The following supporting information can be downloaded at: https://www.mdpi.com/article/10.3390/ijms242115990/s1.
